# Evolution of Heme Peroxygenases: Ancient Roots and Later Evolved Branches

**DOI:** 10.3390/antiox11051011

**Published:** 2022-05-20

**Authors:** Marcel Zámocký, Jana Harichová

**Affiliations:** 1 Laboratory for Phylogenomic Ecology, Institute of Molecular Biology, Slovak Academy of Sciences, Dúbravská Cesta 21, SK-84551 Bratislava, Slovakia; jana.harichova@savba.sk; 2 University of Natural Resources and Life Sciences, Vienna, Department of Chemistry, Institute of Biochemistry, Muthgasse 18, A-1190 Vienna, Austria

**Keywords:** heme thiolate peroxidase, peroxygenase, peroxidase–peroxygenase superfamily, early diverging fungi, Stramenopiles, molecular evolution

## Abstract

We reconstructed the molecular phylogeny of heme containing peroxygenases that are known as very versatile biocatalysts. These oxidoreductases capable of mainly oxyfunctionalizations constitute the peroxidase–peroxygenase superfamily. Our representative reconstruction revealed a high diversity but also well conserved sequence motifs within rather short protein molecules. Corresponding genes coding for heme thiolate peroxidases with peroxygenase activity were detected only among various lower eukaryotes. Most of them originate in the kingdom of fungi. However, it seems to be obvious that these *htp* genes are present not only among fungal Dikarya but they are distributed also in the clades of Mucoromycota and Chytridiomycota with deep ancient evolutionary origins. Moreover, there is also a distinct clade formed mainly by phytopathogenic Stramenopiles where even HTP sequences from Amoebozoa can be found. The phylogenetically older heme peroxygenases are mostly intracellular, but the later evolution gave a preference for secretory proteins mainly among pathogenic fungi. We also analyzed the conservation of typical structural features within various resolved clades of peroxygenases. The presented output of our phylogenetic analysis may be useful in the rational design of specifically modified peroxygenases for various future biotech applications.

## 1. Introduction

Unspecific heme peroxygenases (E.C. 1.11.2.1) represent rather abundant but quite diverse and versatile type of biocatalysts. Oxyfunctionalizations of numerous organic substrates probably represent their most interesting feature, one that has already been investigated in various research fields [1]. However, in contrast to broadly studied cytochrome P450 monooxygenases, they need molecules of hydrogen peroxide for their initial activation [2,3]. This implies that they have to be considered as real peroxidases by following the corresponding typical reaction mechanism, as has been defined previously [4]. Indeed, heme containing peroxygenases have been listed among the four main heme peroxidase superfamilies that arose independently (of each other) during evolutionary history [5]. Currently, this distinct peroxidase–peroxygenase superfamily (previously known in older literature simply as “chloroperoxidases” with another E.C. 1.11.1.10) is in InterPro annotated under #IPR000028 (alternatively also as pfam #PF01328, see http://www.ebi.ac.uk/interpro/entry/Interpro/IPR000028, accessed on 7 April 2022). It is important to note that the name “chloroperoxidase” for this superfamily alone is rather unspecific and not unequivocal. Besides “classical” peroxidase (Reaction 1) and peroxygenase activities (Reaction 2), several described members of IPR000028 can perform also specific haloperoxidase reactions—a common feature with another abundant heme-peroxidase superfamily described in [6]. This type of reaction is summarized in the scheme of Reaction 3.

H_2_O_2_ + 2 AH_2_ → H_2_O + 2 ∗ AH      (Reaction 1)

H_2_O_2_ + RH → H_2_O + ROH        (Reaction 2)

H_2_O_2_ + X^−^ + H^+^ → H_2_O + HOX      (Reaction 3)

2 H_2_O_2_ → 2 H_2_O + O_2_          (Reaction 4)

Moreover, a striking feature of versatile heme peroxygenases is mainly their true catalase activity (Reaction 4, as mentioned in [7]) that turns them into the group of potential enzymatic antioxidants [8], although this specific function of hydrogen peroxide dismutation has not been verified in real physiological conditions yet and thus only some catalase malfunction cannot be ruled out. Heme thiolate peroxidases with monoperoxygenase activity (abbreviated as HTPs) represent a medium-sized oxidoreductase superfamily. Currently, they count for more than 6000 sequenced members with 69 divergent protein architectures (updated according to Interpro database [9] in April 2022). 

For our current phylogenetic analysis, we selected numerous typical HTP members from various fungal and non-fungal phyla, in the latter case predominantly from the taxonomical clade of Stramenopiles, with focus on the phylum Oomycota. We chose typical protein sequences due to estimated frequency of occurrence of corresponding *htp* genes in particular sequenced genomes. Importantly, fungal peroxygenases with already solved 3D structures were included to reconstruct in detail their phylogenetic position with respect to numerous novel sequences of yet-undiscovered proteins that can be sorted and considered for expression and purification in the future. 

The molecular evolution of the whole peroxidase–peroxygenase superfamily based on complete protein sequences was reconstructed with the best evaluated substitution matrix and an efficient maximum likelihood phylogenetic method. After this, we also checked the conserved structural fold and peculiar sequence motifs in newly discovered members mainly from ancestral and non-fungal clades that can be supposed as rather typical for the whole and emerging superfamily with great application potential that is presented and discussed in detail within other contributions of this Special Issue.

## 2. Materials and Methods

### 2.1. Retrieval of Protein Sequences from Databases

All sequences used in this study were retrieved from publicly available databases. Primarily, RedoxiBase—a specific database of divergent oxidoreductases—was chosen to collect the already well annotated *htp* genes [10], but further novel peroxygenase sequences mainly from recently sequenced genomes were also retrieved from GenBank (www.mcbi.nlm.nih.gob/genbank, accessed on 7 April 2022) and UniProt (www.uniprot.org, accessed on 7 April 2022) databases with the help of corresponding Protein-Blast searches in each particular database. Only full-length sequences with clearly resolved exons and introns representing well-described fungal taxa were included in this phylogenetic analysis.

### 2.2. Multiple Sequence Alignment 

Complete protein sequences of the selected 174 members belonging to the peroxidase–peroxygenase superfamily are listed in Table S1 with corresponding accession numbers, origin, and abbreviations used for each analyzed member. These protein sequences were aligned using the Muscle program [11] implemented in MEGA-X phylogenetic suite [12]. Optimized alignment parameters were gap open −0.8, gap extend −0.05, and hydrophobicity multiplier 0.9, and the output was inspected and manually refined with GeneDoc software version 2.7.000 (www.psc.edu/biomed/genedoc, accessed on 3 March 2022). Putative signal sequences of here-analyzed peroxygenases with detection of Sec/SPI cleavage sites allowing the secretion were predicted with SignalP 6.0 server [13] by applying the default parameters for eukaryotic proteins.

### 2.3. Reconstruction of Evolutionary Relationships

Phylogenetic relationships within obtained multiple sequence alignment of 174 peroxygenases were reconstructed using MEGA-X suite [12]. The maximum likelihood method was chosen with the application of the Le_Gascuel substitution model [14] (abbreviated LG + G + I) as the statistically proven model with the lowest Bayesian information criterion score among the 57 analyzed models. Gamma-distributed substitution rates with the presence of invariant sites and 4 categories (+G, parameter = 1.1154) were used for this reconstruction. Partial deletion with site coverage cut off 75% and very strong branch swap filter were selected. This means that 239 alignment positions were used for the conducted phylogenetic reconstruction and 1000 bootstrap replicates were applied. The resulting phylogenetic tree was then exported in the Newick format in iTOL server (https://itol.embl.de, accessed on 21 February 2022) [15] where it was arranged in circular form, and obtained bootstraps/metadata values in the range of 0.51 to 1 were visualized on the branches. Afterwards, it was exported as a full image in a scalable vector graphics file.

### 2.4. Analysis of Transcription Regulatory Regions of HTP Genes

Transcription regulatory elements embedding HTP genes together with the position of introns were analyzed with the FGENESH 2.6 program of the Softberry suite (www.softberry.com, accessed on 13 May 2022) [16]. Corresponding DNA sequences were analyzed by using HMM-based gene structure prediction with gene-finding parameters based on the taxonomically most closely related organisms already present in the Softberry database.

### 2.5. Comparison of 3D Structures of Various Heme Peroxygenases

The structures of heme peroxygenases mainly from clades with none known X-ray structure were modelled with PHYRE2 protein fold recognition server [17] by using the intensive mode of analysis with the batch submission approach. Obtained homology models were rendered in PyMOL (https://pymol.org, accessed on 7 April 2022) for investigating the diversity in structures of yet putative peroxygenase sequences mainly from the basal clades of the here-reconstructed superfamily. 

## 3. Results

### 3.1. Reconstructed Phylogenetic Tree of the Peroxidase–Peroxygenase Superfamily

**Figure 1:**
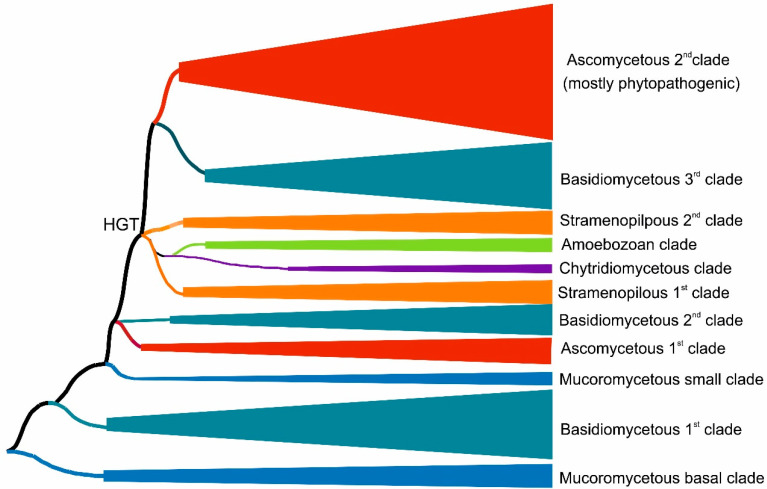
Global evolutionary tree for the peroxidase–peroxygenase superfamily obtained with the maximum likelihood method within the MEGA X suite [12] by using the Le_Gascuel model of amino acid substitution [14] and 1000 bootstrap replications. Only the distribution of phyla in major grouped clades are shown here schematically. Details of the topology for particular HTP sequences are presented in Figure 2.

**Figure 2:**
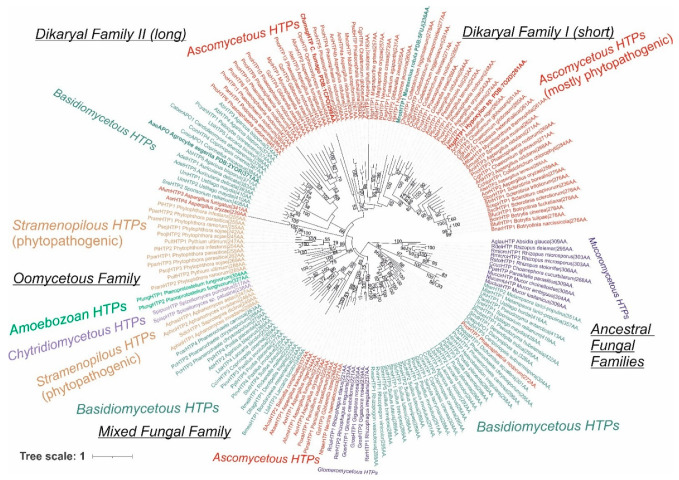
Detailed reconstructed evolutionary tree in circular form for the analyzed 174 sequences of the peroxidase–peroxygenase superfamily obtained with the maximum likelihood method of the MEGA X suite [12]. The Le_Gascuel model of amino acid substitution [14] with gamma-distributed substitution rates and with the presence of invariant sites (LG + G + I) as the statistically best proven model was applied for this reconstruction. Bootstrap analysis was performed with 1000 replications, and only values in the interval 51–100% (majority-rule consensus) are presented next to corresponding branches. All abbreviations of used sequences are listed in Table S1.

We reconstructed the evolutionary relationships within the selected set of 174 full-length protein sequences coding for divergent heme peroxygenases (listed in Table S1) with the maximum likelihood method by applying the best proven amino acid substitution model obtained with lowest BIC (Bayesian information criterion) score. The same output is depicted in Figure 1 and Figure 2 in different presentation modes. Whereas Figure 1 presents a condensed global view of obtained phylogeny, in Figure 2, all particular details in resolved branches are shown. Although the dominant portion of determined branches is occupied by fungal representatives, non-fungal sequences also form important parts of this phylogenetic tree and reveal a broad dissemination of *htp* genes among early diverging eukaryotes.

#### 3.1.1. Fungal Representatives of Heme Peroxygenases

Fungal heme peroxygenases have already been intensively investigated for their diverse reactivity, and there are several representatives with already solved 3D structures [18,19,20,21]. The phylogenetic distribution of most *htp* genes corresponds with a generally accepted evolutionary distribution of all known fungal phyla [22] with a small exception of the position for chytridiomycetous *htp* representatives (Figure 1 and Figure 2) that are still rather scarce in comparison with numerous known dikaryal sequences. In basal clades of this tree resolved with the maximum likelihood approach, heme peroxygenase sequences from early diverging fungi [23] occur (Figure 1—lower part, and Figure 2—right in the middle). Namely, numerous *htp*-coding genes from the genera *Mucor* and *Rhizopus* were discovered in recent genome sequencing projects, e.g., [24,25]. In comparison with short versions of ascomycetous and basidiomycetous peroxygenases (e.g., PDB code: 7O2D), they have longer coding sequences that are however shorter if compared with long dikaryal HTPs (e.g., PDB code: 1YOR; for details, see Figure 2 and Table S1). The calculated average length for this basal *Mucor* clade is 301 amino acids, and the average molecular mass (with incorporated heme) is 35 kDa. According to UniProt annotations (links in Table S1), they all contain at least one N-terminal transmembrane α-helix. This would suggest that ancient HTPs were probably middle-sized proteins and that they were integral components of ancestral fungal membranes. 

Recent large phylogenomic analysis of 136 concatenated genes (without involvement of oxidoreductases) in 40 diverse fungal genomes demonstrated the evolutionary basal position of Mucor and Rhizopus clades against all dikaryal genomes [26]. The here-obtained phylogenetic results are in accordance with this general output, revealing a basal HTP clade for the whole superfamily originating from Mucoromycota. 

However, the phylogenetic distribution is more complicated if following in the direction to direct descendants of mucoromycetous HTPs (Figure 2). We can observe the occurrence of a large clade formed mostly with basidiomycetous HTP representatives. Later in the evolution, a second, smaller glomeromycetous HTP clade appeared. These two clades can be described as early descendants from the ancestral *Mucor* clade. It is likely that multiple horizontal gene transfers (HGTs) took place at this evolutionary stage. The clade formed by dominantly basidiomycetous representatives contains sequences between 272 and 428 amino acids long (in average 316 AA), but these are almost exclusively uncharacterized proteins from recent genome sequencing projects and so their detailed characterization is urgently needed. Most of the representatives within early diverging dikaryal clades stem from classes Pucciniomycetes and Ustilaginomycetes that are known as dangerous phytopathogens [27]. Even one short ascomycetous phytopathogenic representative that is probably a product of a more recent HGT can be found among the clades of this subfamily. 

Glomeromycetes were considered as a specific class of the phylum Mucoromycota [26], but more recent analyses position them in a separate phylum Glomeromycota [22]. A distinct subfamily of glomeromycetous HTP (Figure 2 lower part) can be observed in this reconstruction. It contains only short sequences with an average size of 230 amino acids that corresponds to only 26.8 kDa protein. Some of its representatives were annotated as having coiled coil motif in their structures at C-termini. As Glomeromycota are known as obligate symbionts of land plants, the corresponding HTP proteins may have obtained novel functions in comparison with above discussed predecessor *Mucor* clades. 

Next, descendant clades in the reconstructed phylogenetic tree were formed by a small subfamily of ascomycetous short representatives (in average 265 amino acids long), followed by clades of basidiomycetous HTPs with an average size of 279 amino acids. They can be together understood as a mixed fungal family formed directly after evolution from ancestral fungal HTP families. In both observed clades, many sequences from phytopathogenic representatives were included, and they were probably shared by an HGT event between evolutionarily unrelated fungi. Further descendant clades were formed by non-fungal representatives, which were analyzed separately in Section 3.1.2. 

After segregation of non-fungal HTP genes in their own clades, the evolution within this superfamily also continued further among fungal basidiomycetous and mainly ascomycetous clades that were less intermixed, as is obvious from the comparison of the upper and lower parts of Figure 2. This region of reconstructed phylogenetic tree contains several members of the peroxidase–peroxygenase superfamily that were investigated in the most detail, e.g., [18,20,21]. It is important to mention that two distinct ascomycetous and two distinct basidiomycetous proteins with experimentally solved 3D structures are located here (see Figure 2 and Table S1 for details on their PDB codes). 

Two HTP families previously defined as “II—long” and “I—short” [28] are located in the upper parts of the reconstructed tree. From the here-presented phylogenomic reconstruction, it is obvious that Family II—long is formed by both basidiomycetous and ascomycetous representatives, whereas Family I—short is formed predominantly by diverse ascomycetous proteins. There are only rare exceptions that occurred probably via a late HGT event from the Family I ascomycetous gene into the basidiomycetous genome. Sequence analysis showed that Family II according to the here-presented sequences has an average length of 402 amino acids, but Family I revealed on average only 265 amino acids, so there is a striking difference between these two distinct types of dikaryal sequences. On the other hand, Family I has a comparative length with the mixed fungal family mentioned above. Most of the later evolved clades of Ascomycota sequences from Family I are occupied by HTP representatives from dangerous phytopathogenic fungi, but also several other representatives (e.g., MtherHTP) from thermotolerant fungi occur in last segregated branches of Family I.

#### 3.1.2. Non-Fungal Representatives of Heme Peroxygenases and Their Phylogenetic Position

Besides the numerous phylogenetically grouped fungal sequences, it is obvious that also distinct clades of non-fungal heme thiolate peroxidases occur in the reconstructed evolutionary tree. From the details of Figure 2, it is apparent that well-segregated clades in the left middle part can be understood as a separate HTP family that is named the oomycetous peroxygenase family due to the origin of the majority of its representatives. Due to numerous previous phylogenomic analyses, it is now generally accepted that the phylum Oomycota does not belong to the kingdom of fungi [29]. These microorganisms are part of a large taxonomical clade of Stramenopiles, also known as heterokonts, that differ from classical fungi mainly by the presence of differently arranged flagella. All HTP sequences of this family very likely arose from ancestors among basidiomycetous HTP of the already defined mixed fungal family via a horizontal gene transfer (HGT). The average size of representatives from this dominantly non-fungal and exclusively non-dikaryal family is 276 amino acids and 30.9 kDa—that is, nearly the same as for their direct basidiomycetous predecessors in neighboring clades, supporting the HGT hypothesis. Quite distinct and most abundant among the here-presented oomycetous peroxygenase family are two well-separated clades. Representatives of genera *Aphanomyces* and *Saprolegnia* known mostly as fish and crayfish pathogens and also as saprotrophs form the first clade. For example, SdicHTP1 comes from *Saprolegnia diclina*, known as a devastating parasite of amphibians [30], but its physiological function is yet unknown. The second oomycetous clade is dominated with HTP sequences from the dangerous phytopathogenic genus *Phytophthora*, known to cause enormous losses in many agricultural products and forests worldwide [31]. Their real physiological function has also not been addressed yet. However, also two chytridiomycetous HTPs were found in clades of this taxonomically rather heterogenous family. These two unique sequences do not cluster with mucoromycetous counterparts as would be expected from a general model of fungal phylogeny [22]. Thus, HTP genes of this specific family were additionally transferred into the genomes of Spizellomyces by a later HGT event, probably from some oomycetous predecessors. 

Surprisingly, there are two novel sequences of this family discovered in the genome of *Protostelium aurantium* that belong to the protosteloid Amoebozoa [32]. These unique predator microorganisms are phylogenetically positioned far from the kingdom of Fungi and also far from Stramenopiles. Trophozoite stages of these protists have a remarkable fungivorous lifestyle with the generation of reactive oxygen species [33]. This fact would potentially explain a late HGT event of peroxygenase genes from some ancient fungal prey into the amoebozoan predator genome that might represent a positive evolutionary advantage for descendants of the *Protostelium* species. 

### 3.2. Prediction of Signal Sequences in Heme Peroxygenases

In Table 1, the analysis of signal sequences obtained from the SignalP 6.0 server is summarized for typical HTP sequences of each resolved evolutionary clade of this superfamily. It is obvious that not all heme peroxygenases are secreted outside of the cells, and this is in evident contrast with, e.g., typical fungal lignin, manganese, and versatile peroxidases [34], but also with fungal Hybrid B heme peroxidases discovered recently [35]. Interestingly, most of the mucoromycetous HTPs positioned at the ancestral roots of the superfamily are not targeted to the secretome. Therefore, the output of the presented phylogenetic analysis supports a hypothesis that ancestral peroxygenase was probably an intracellular enzyme [28]. Moreover, the most ancestral clade of basidiomycetous HTPs (lower part on the right of Figure 2) and glomeromycetous sequences (lower part of Figure 2) revealed a similar intracellular location. During the further evolution of the descendant clades of this tree, the portion of secreted peroxygenases increased. In the oomycetous family, the majority of peroxygenase isozymes have already been predicted as secreted, and this can correspond with their mostly phytopathogenic lifestyle. However, only two sequenced amoebozoan HTPs that also belong to this family were predicted as intracellular. More amoebozoan sequences of this family are needed to make a reliable decision about their subcellular location.

Contrary, in the (fungal) dikaryal Family I and II, a vast majority of presented sequences including, e.g., the intensively investigated aromatic peroxygenases, was predicted for the secretome. However, if some peroxygenase isozymes (originating from the same or very similar organisms) are secreted outside of the cell while a remaining portion of HTP isozymes are located in the cytosol, they probably need to fulfill a diverse function in the cellular metabolism. There is a good example for five AbHTP paralogs that were all sequenced from the genome of a well-known mushroom *Agaricus bisporus* (Table 1). Only three of them are predicted for the secretome, i.e., they are targeted for an extracellular location, and the remaining two are intracellular. No detailed subcellular location of these non-secreted HTP variants in, e.g., mitochondria or peroxisomes have been able to be detected so far.

### 3.3. Gene Architecture of HTP Genes

**Figure 3:**
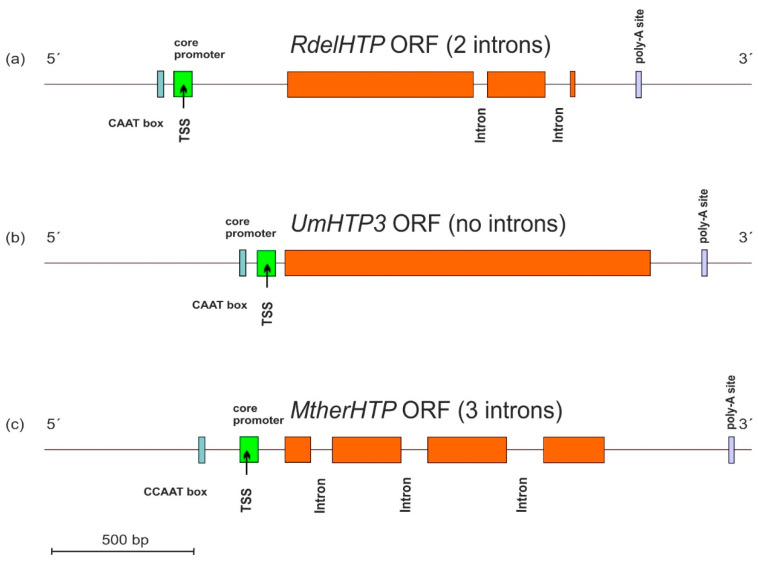
Typical gene architecture of (**a**) mucoromycetous, (**b**) basidiomycetous, and (**c**) ascomycetous HTP gene with detection of the core promoter region (labelled green), CCAAT box (cyan), and poly-A site (violet). TSS—transcription start site. Orange—HTP coding regions. The distribution of introns and position of regulatory elements is drawn to scale according to predictions from FGENESH [16].

Typical architecture of sequenced HTP genes including the promoter region and also the 3′ non-coding region in the genomic DNA of three selected fungi from their available genomes was analyzed with program FGENESH [16], and the results are presented in Figure 3.

These three presented genes originate from distantly related fungal phyla of Mucoromycota, Basidiomycota, and Ascomycota and reveal differences in the location of cis-regulatory elements in corresponding DNA regions mainly in the position of CAAT boxes responsible for specific binding of the transcription factors. Apparently, there are also significant differences in the number and topology of introns between presented DNA sequences that probably reflect easy movement of rather short introns along HTP genes within divergent fungal genomes. 

### 3.4. Conserved Protein Sequence Motifs in Aligned Heme Peroxygenases

**Figure 4:**
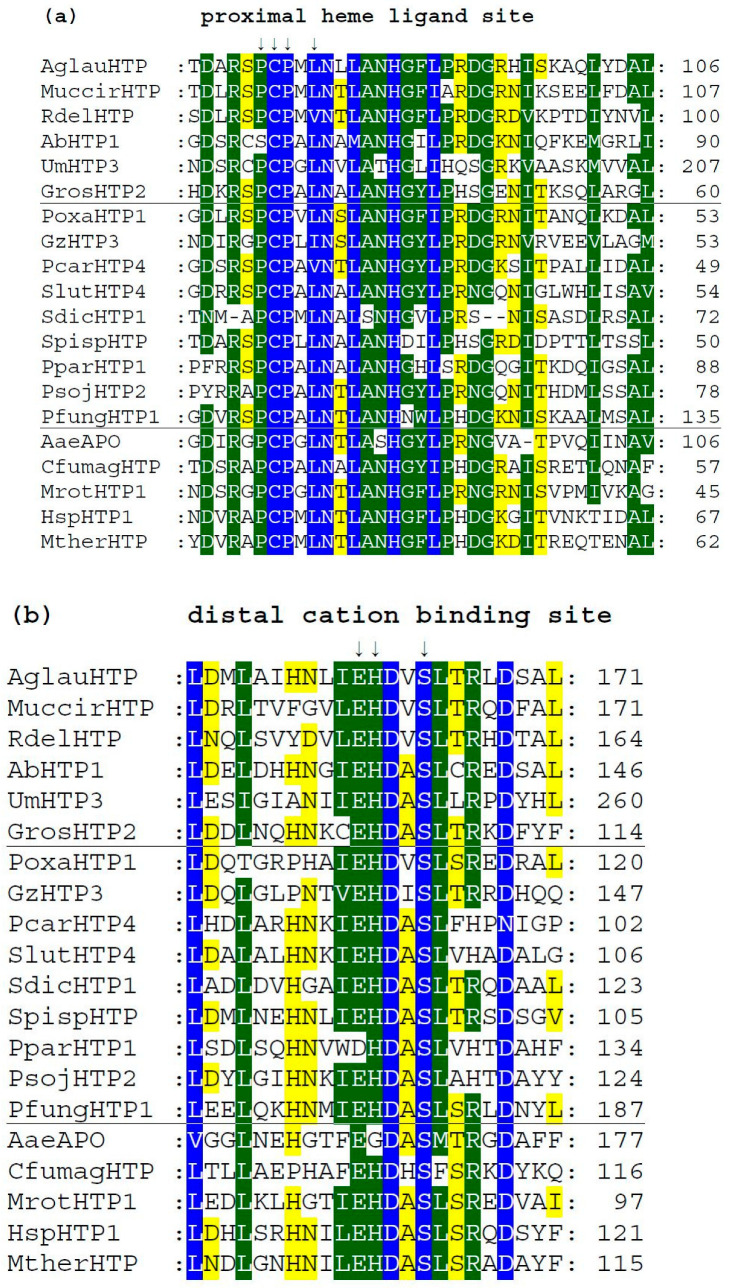

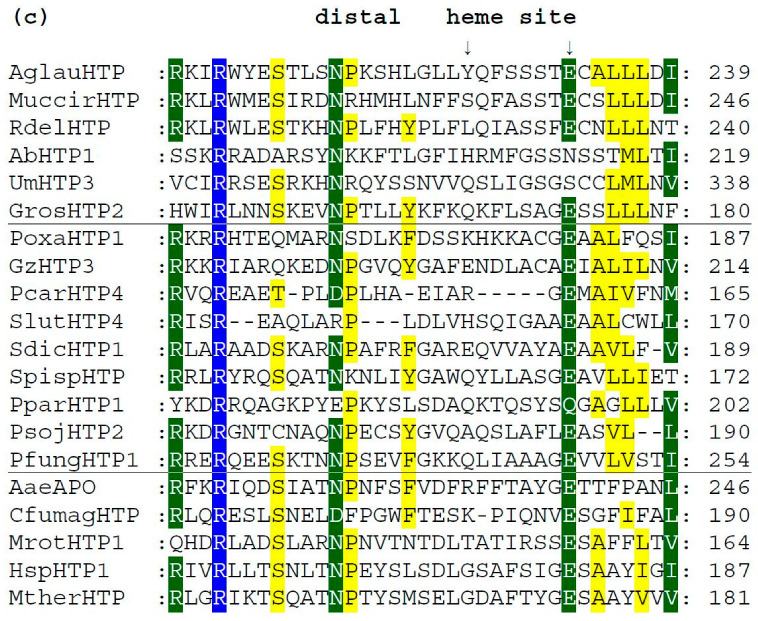
Multiple sequence alignment of 20 selected heme thiolate peroxidases. Only three highly conserved regions from the obtained muscle protein alignment [11] are shown here. Complete sequence alignment in FASTA format is provided in Figure S1. Regions shown: (**a**) sequence motif around the proximal ligand of heme; (**b**) motif with amino acid ligands responsible for the binding of a metal cation; (**c**) distal site of the prosthetic heme group. Abbreviations of all sequence names are explained in Table S1, together with their accession numbers in databases. Color scheme: blue > 95%, green > 75%, and yellow > 50% of overall conservation.

We analyzed typical protein sequence features in various clades of the obtained phylogenetic tree for the whole peroxidase–peroxygenase superfamily. Important parts of the corresponding multiple sequence alignment were selected within 20 typical protein sequences of the whole superfamily. They are presented in three separate panels of Figure 4, showing regions of proximal heme ligand, metal cation binding site, and distal region around the heme, respectively. Three different background colors reflect the levels of the overall sequence conservation at each alignment position. 

Among the presented sequences, four non-fungal HTP representatives (discussed in Section 3.1.2.) were included to reveal their conserved features and possible differences. Among the aligned fungal peroxygenases, a representative from thermophilic fungus (MtherHTP) is also shown, but no significant difference with well-known mesophilic counterparts could be revealed in these three regions. Conserved sequence patterns of all heme thiolate peroxidases important for their typical function are located mainly on the N-terminus of presented protein sequences. Complete (full length) sequence alignment is presented in Figure S1 in FASTA format. From the details shown in Figure 4a, the most typical feature of these enzymes is shown—a highly conserved pattern that includes the proximal heme ligand as invariant cysteine embedded by two prolines [20]. The corresponding consensus sequence around the proximal heme ligand is **D-X-R-X-P-C-P-X-L/V/I-N**. This means that the surrounding of these essential three amino acids also reveals a high level of conservation obvious also from [18].

There is a rare exception within this otherwise highly conserved amino acid triad in basidiomycetous intracellular AbHTP1 (upper part of the alignment), where the first proline of the mentioned proximal heme ligand is exchanged to a serine. Even a second extra cysteine is aligned in its close neighborhood, but this is just a hypothetical protein, so no conclusion on its real function can be made at the moment.

Further downstream, the conserved motif with the consensus of **I/V/L-E-H-D-X-S** on the distal side of heme (Figure 4b) contains amino acid ligands that are involved in the binding of metal cations. It is interesting to mention that magnesium ions are found at this site in known structures of AaeAPO, MrotHTP1, and HspHTP1. Only in CfumagHTP were manganese ions described at comparable position as obvious from corresponding pdb file 1CPO. For this first ever known 3D structure of a heme peroxygenase [18], the position of His 105 was found to be essential for manganese cation binding. Indeed, this position is highly conserved in Figure 4b (labelled with an upper arrow). Only AaeAPO has a substitution to Gly at a corresponding position, and it is known to bind magnesium instead of manganese ions [19]. Curiously, other sequences of peroxygenases with known 3D structures (with PDB codes 5FUJ and 7O2D [20,21]) are referred to as also binding magnesium although containing His in corresponding positions. Nonetheless, in each of these cases, binding of metal cations at the distal site of heme stabilizes the conformation of the essential heme prosthetic group in active centers of various HTPs. 

In Figure 4c, sequence patterns on the distal site of heme further downstream to C-terminus are presented, but obviously in this region, a lowest level of overall conservation can be observed. The already mentioned intracellular AbHTP1 revealed significant differences in this region also, but the principal question is whether the replacement of glutamate to asparagine in this case is possible in terms of fulfilling the proposed catalytic function of peroxide bond cleavage. This is achieved during compound I formation, which is known as an oxidized reactive intermediate of all heme peroxidases [18,20]. Nonetheless, other HTP sequences also reveal a rather high level of diversity in this region that may reflect their flexible reactivity.

### 3.5. Structural Comparisons of Conserved Protein Architecture

**Figure 5:**
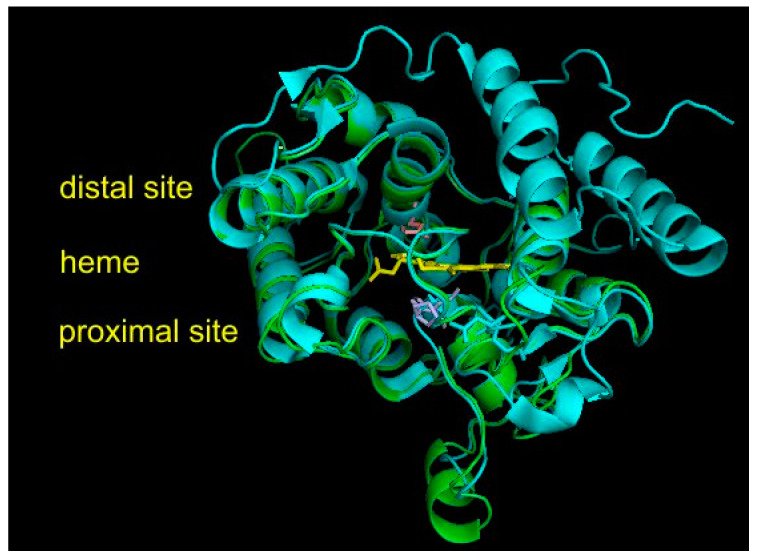
Structural overlay between AaeAPO (peroxygenase from *Agrocybe aegerita* data obtained from PDB file 2YOR [19]) with a homology model for RdelHTP (peroxygenase from *Rhizopus delemar*) obtained with Phyre^2^ [17]. Color scheme: AaeAPO—cyan, RdelHTP—green, prosthetic heme group—yellow, essential amino acid residues from RdelHTP (shown in the alignment of Figure 3) on the proximal site of heme—violet, amino acid residue on the distal site—pink. Rendered with PyMOL.

The experimental 3D structure of heme thiolate peroxidases has already been solved for four members of this superfamily that, however, all belong either to Family I (short) or to Family II (long) of the fungal subkingdom Dikarya. They are included in Table S1, together with their PDB accession codes for overview. However, no experimental structure is known yet for any member of the oomycetous family; the mixed fungal family; and, most importantly, the ancestral fungal families (cf. Figure 2). Therefore, homology modeling on Phyre^2^ server [17], focused mainly on HTP representatives from ancestral fungal clades, was performed to reveal the level of conservation of the structural fold typical for possible roots of this superfamily. For all 10 representatives originating in the ancestral mucoromycetous clade (Figure 2), confidence of 100% with pairwise alignment coverage ranging between 71 and 84% was achieved. The best model, namely, for RdelHTP also analyzed in the alignment of Figure 4, was taken from the modeling server and is presented in Figure 5 as a structural overlay with the experimentally obtained 3D structure of AaeAPO subunit A with PDB code 2YOR [19] originating from the mushroom *Agrocybe aegerita*.

Interesting facts can be drawn from this structural figure. Although the overall architecture of the active center is well preserved during evolution from Mucoromycota towards Basidiomycota and also the distal and proximal sites near the heme group look very similar, there are fine differences located mainly on the surface of overlaid proteins. Namely, there are two short α-helices present in the mucoromycetous HTP pointing towards the proximal site of heme that are obviously not present in basidiomycetous peroxygenases. On the contrary, two long α-helices on the right distal site of basidiomycetous peroxygenase are not seen in the evolutionarily distantly related protein from a basal fungal lineage. These fine differences may reflect a different folding stability and probably also some differences in the reactivity. However, for RdelHTP and related mucoromycetous representatives of the basal peroxygenase family, these structural aspects need to be verified experimentally.

## 4. Discussion

Heme peroxygenases were already intensively applied in synthetic organic chemistry and pharmaceutical technology to produce valuable compounds via catalytic oxygenation, oxidation, and demethylation [1]. However, their natural role and real physiological function still remain unknown. Recently, a phylogeny comprising 113 fungal unspecific peroxygenases stemming only from species of Basidiomycota and Ascomycota was presented [36]. A more broadly formulated phylogenetic reconstruction and sequence analysis presented in this contribution also covers the basal fungal lineages, namely, Mucoromycota, Glomeromycota, and Chytridiomycota, that are still under-represented in sequence databases. This more comprehensive approach shall instigate new directions of research on heme thiolate peroxidases. A more detailed approach on the reconstruction of UPO/HTP phylogeny was described in [37]. However, this was performed only with the rapid neighbor-joining method, and no bootstrap values were given. 

A unique online database for peroxygenases was established recently [38], allowing for phylogenetic analysis with the same maximum likelihood approach, as described in Section 2.3. Thus, a more extensive phylogeny mainly including numerous sequences from basal fungal lineages will be possible in the future. However, even among the already quite well discovered members of dikaryal long and short HTP families there are still numerous sequences, foremost in dangerous phytopathogens available in genomic databases with yet putative properties. Besides genomics and proteomics, a priority shall be set in the near future on detailed transcriptomic analysis of mainly those HTPs produced by pathogenic and also non-pathogenic fungi with application of RT-qPCR methodology. It shall be mainly investigated as to whether specific mRNAs of heme peroxygenase genes are stimulated in response to attack of the particular plant host or in a reaction to host defense mechanisms, mainly producing oxidative burst [39] in a similar way as was shown recently, e.g., for hybrid B heme peroxidases in rice blast fungus [40]. 

Besides studying genes and proteins produced by numerous ascomycetous and basidiomycetous pathogens, still nothing is known about the occurrence and production of HTPs from the Oomycetous family. Very probably, in these phytopathogens, heme peroxygenases are also involved in a broad response to the defense of hosts producing ROS. In a completely different approach, the fungivorous amoeba *Protostelium aurantium* contains two peculiar HTP sequences that also urgently need a comprehensive investigation of their structure and reactivity as this may contribute to our understanding of peroxide metabolism of this unusual amoeba [33]. 

By going back to the roots of the reconstructed evolutionary tree, we notice that at the proteomics and expressed protein levels, none of putative heme thiolate peroxidases are currently known from the division of *Glomeromycota* with very ancient roots and already over 230 described species. Their trophic interactions are still under intensive discussion [41], and the discovery of corresponding HTP enzyme reactivity for at least a few typical members may shed light to their still rather unknown metabolism. At the supposed roots of the whole peroxidase–peroxygenase superfamily, representatives from Mucoromycotina that are classified as basal fungal lineages are positioned. They are still referred to as hypothetical proteins and need utmost attention for future research on genomic, transcriptomic, and proteomic levels. Their typical reactivity based mainly on oxyfunctionalization with various physiological and non-physiological substrates can be inspiring in the future for various promising applications outlined to some extent in [28,38].

## 5. Conclusions

In this contribution, we made an attempt at reconstructing a reasonable route of phylogenetic relationships within the entire peroxidase–peroxygenase superfamily constituted by heme peroxygenases from ancient roots towards later evolved clades. Further, we have shown that mainly the non-fungal and ancestral fungal clades still contain plenty of interesting and yet unknown candidates for intensive future research on their molecular structure, stability, and physiological function.

## Figures and Tables

**Table 1:** Prediction of N-terminal signal peptide (Sec/SPI) in selected sequences of heme peroxygenases (HTP) for the Sec pathway over the endoplasmic reticulum membrane. Performed with [13] by using the default parameter setting for eukaryotic protein sequences.

Sequence Abbreviation ^1^	Signal Sequence Length (AA)	Prediction Probability
AbHTP1 (B)	0	
AbHTP2 (B)	0	
AbHTP3 (B)	20	0.9781
AbHTP4 (B)	20	0.9806
AbHTP5 (B)	20	0.9879
BcinHTP (A)	19	0.7056
CaberAPO1 (B)	24	0.9767
CcinHTP1 (B)	0	
CcinHTP2 (B)	0	
CcinHTP3 (B)	0	
CcinHTP4 (B)	25	0.9377
CgHTP2 (A)	17	0.9762
DaspAPO1 (B)	0	
FagHTP1 (A)	17	0.9763
GcerHTP1 (G)	0	
GzHTP1 (A)	18	0.9795
GzHTP2 (A)	14	0.9708
GzHTP3 (A)	0	
GzHTP4 (A)	0	
GzHTP5 (A)	20	0.9917
HjHTP1 (A)	19	0.7098
MgrHTP1 (A)	24	0.5823
MoHTP1 (A)	24	0.6147
NhaeHTP (A)	0	
PcyanHTP1 (B)	20	0.9847
PfungHTP1 (Am)	0	
PfungHTP2 (Am)	0	
PficHTP1 (A)	18	0.8108
PparHTP1 (S)	24	0.9815
PparHTP2 (S)	17	0.9860
PparHTP3 (S)	0	0.5008
PramHTP1 (S)	17	0.9752
RdelHTP (M)	39	0.8967
RmicroHTP1 (M)	0	
RstolHTP (M)	0	
SborHTP1 (A)	17	0.9894
SsclHTP1 (A)	19	0.6837
SpipunHTP (C)	0	
ThetHTP1 (A)	17	0.9738
UmHTP1 (B)	22	0.9940
UmHTP2 (B)	22	0.9834
UmHTP3 (B)	0	

^1^ For abbreviations of all here-presented sequences, refer to Table S1. Assignment to phyla: (A)—Ascomycota, (Am)—Amoebozoa, (B)—Basidiomycota, (C)—Chytridiomycota, (G)—Glomeromycota, (M)—Mucoromycota, (S)—Stramenopiles—here only Oomycota sequences.

## Data Availability

All used data are contained within the article and Supplementary Materials.

## Supplementary Materials

The following are available online at https://www.mdpi.com/article/10.3390/antiox11051011/s1, Figure S1: Multiple sequence alignment of 20 heme thiolate peroxidases. Table S1: Overview on abbreviations of all used peroxygenase sequences with their accession numbers in sequence databases.
