# Supplementary material for: Evolution of Heme Peroxygenases: Ancient Roots and Later Evolved Branches
Source: Antioxidants (Basel). 2022 May 20;11(5):1011. doi: 10.3390/antiox11051011 (PMC9138132; doi:10.3390/antiox11051011)
Supplement: Supplementary file 1 [file antioxidants-11-01011-s001.zip › Figure S1_20 HTPs aligned.pdf]

```
                      *        20         *        40         *        60         *        80
AglauHTP_A : M----------------------------GKRKSGAKQQQQQQQQQRRSQWSFKR--------------------LMVGA :  32
MuccirHTP_ : M---------------------------SKLENKTLFPKTPPQKKSKSAASTIIS--------------------VILGL :  33
RdelHTP_Rh : M-----------------------------------TEKVKKNQRVSIKKSRIGS--------------------LLLGL :  25
AbHTP1_Aga : M-----------------------------------FILFKIFHDIAFFSWDLGL------------------------- :  20
UmHTP3_Ust : MTFPTRPSTPSYSMPATKTKVEAKGLRDESHTMHKSNSSMIRLTAQLMQQNRPQVAVNIALFFIFLVDFWLTSFNVFHSA :  80
GrosHTP2_G : M------------------------------------------------------------------------------- :   1
PoxaHTP1_P : M------------------------------------------------------------------------------- :   1
GzHTP3_Gib : M------------------------------------------------------------------------------- :   1
PcarHTP4_P : M------------------------------------------------------------------------------- :   1
SlutHTP4_S : M------------------------------------------------------------------------------- :   1
SdicHTP1_S : M-----------------------------------------------RPTSLQM------------------------- :   8
SpispHTP_S : M------------------------------------------------------------------------------- :   1
PparHTP1_P : M-------------------------------VSLGTSLVAAAVAVASFASPSSS------------------------- :  24
PsojHTP2_P : M-------------------------------VSFTRSIAVAAIATISSAWALQE------------------------- :  24
PfungHTP1_ : M---------------SSWKASIPYRPAHLLIEEDFGYSYVCISDFQGKRSWSVSPRTRESFFVWPPFTGQKPQMKTAFI :  65
AaeAPO_Agr : M------------------------------------KYFPLFPTLVFAARVVAF--------------------PAYAS :  24
CfumagHTP_ : E------------------------------------------------------------------------------- :   1
MrotHTP1_M : S------------------------------------------------------------------------------- :   1
HspHTP1_Hy : M-------------------------------------------KSLSFSLALGF------------------------- :  12
MtherHTP_M : M------------------------------------------------------------------------------- :   1
             m
```

```
                      *       100         *       120         *       140         *       160
AglauHTP_A : ILGFLVIQVIRIELQAA-GSTKTPD-----------------------------------------EWRALVK------- :  63
MuccirHTP_ : IAAYLVSAIINIEVTGR-RGLKSTA-----------------------------------------EWKELIK------- :  64
RdelHTP_Rh : FTTFTVLGAILLEADSRSKGIKSPE-----------------------------------------QWYEIMK------- :  57
AbHTP1_Aga : ---TFVNLVLPNRPVGKVTPEGHPG-----------------------------------------Y------------- :  43
UmHTP3_Ust : LIKLGVINPLPENCVPPAFSPTSPPSFAQWPLTHIYCRLIHIYNFVTSYSEDERLHHDLYCRKPKNDWIRDWAYWDGIGH : 160
GrosHTP2_G : ---------NSFRLLHSIKRYSMS-------------------------------------------------------- :  16
PoxaHTP1_P : ---------------PSQLQALE--------------------------------------------------------- :   9
GzHTP3_Gib : ---------------ASSIQPLT--------------------------------------------------------- :   9
PcarHTP4_P : ---------------------SSSG------------------------------------------------------- :   5
SlutHTP4_S : ----------------STPNWTCPP------------------------------------------------------- :  10
SdicHTP1_S : ---AAALAVLATSARRFASYASNPP-----------------------------------------P------------- :  31
SpispHTP_S : ---------------------TSTT-----------------------------------------A------------- :   6
PparHTP1_P : -----RVLDPNKLVVGEFFKPSGHI-----------------------------------------V------------- :  45
PsojHTP2_P : ---------------GEYYRPTTSE-----------------------------------------A------------- :  35
PfungHTP1_ : FALLFLAAAAYPRKNGKIVFPAYDD-----------------------------------------D------------- :  91
AaeAPO_Agr : LAGLSQQELDAIIPTLEAREPGLPP-----------------------------------------GPLENSSAKLVNDE :  63
CfumagHTP_ : --------------PGSGIGYPYDN-----------------------------------------N------------- :  13
MrotHTP1_M : -------------------------------------------------------------------------------- :   -
HspHTP1_Hy : ---------------GSTLVYSAPS-----------------------------------------P------------- :  23
MtherHTP_M : --------RASVLPVLIAISPALAG------------------------------------------------------- :  18
```

```
                      *       180         *       200         *       220         *       240
AglauHTP_A : QRPYA----RLPTDARSPCPMLNLLANHGFLPRDGRHISKAQLYDALVLVGAPPTITYGFLTTVVYR------------- : 126
MuccirHTP_ : EHPYQ----RKDTDLRSPCPMLNTLANHGFIARDGRNIKSEELFDALMLMGAPPTVTVVILNYVFSQ------------- : 127
RdelHTP_Rh : SHPYE----RKLSDLRSPCPMVNTLANHGFLPRDGRDVKPTDIYNVLSLLRIPPVFSVGIIAFAYSQ------------- : 120
AbHTP1_Aga : GGKWPEYIPPKEGDSRCSCPALNAMANHGILPRDGKNIQFKEMGRLIRTTYNFSSSFCYFVPHYAAE------------- : 110
UmHTP3_Ust : KGEWGAPYPARDNDSRCPCPGLNVLATHGLIHQSGRKVAASKMVVALSRAFNIAPTMALQLYSPLFP------------- : 227
GrosHTP2_G : AHVWQ---GPGTHDKRSPCPALNALANHGYLPHSGENITKSQLARGLQEGLNASSLLANFLAYGGFN------------- :  80
PoxaHTP1_P : KGEFR---PADSGDLRSPCPVLNSLANHGFIPRDGRNITANQLKDALQRIGTGVDIRHTLVNRAFTV------------- :  73
GzHTP3_Gib : KGVYA---PSGPNDIRGPCPLINSLANHGYLPRDGRNVRVEEVLAGMNAVGLSKPLGAAFANPIFQERAPSKFHDDQVTQ :  86
PcarHTP4_P : SHAFC---PPMEGDSRSPCPAVNTLANHGYLPRDGKSITPALLIDALTQCYRLSKPLAWILAYGGLL------------- :  69
SlutHTP4_S : AHPFI---PATQGDRRSPCPALNALANHGYLPRNGQNIGLWHLISAVQEVYNLSFILAAVLALAGVL------------- :  74
SdicHTP1_S : FQPWS---PTDPTNM-APCPMLNALSNHGVLPRS--NISASDLRSALGALQCDSLIQRILSRDSVMA------------- :  92
SpispHTP_S : HPQWK---PAGKTDARSPCPLLNALANHDILPHSGRDIDPTTLTSSLRKAGIDPGISDRLVSITMGM------------- :  70
PparHTP1_P : SGFFN---TTA-PFRRSPCPALNALANHGHLSRDGQGITKDQIGSALKSVLNQGDDAVVALLASLPE------------- : 108
PsojHTP2_P : SGVYG---TTA-PYRRAPCPALNTLANHGYLPRNGQNITHDMLSSALQKVYNIGPGVAALLVAALPD------------- :  98
PfungHTP1_ : EHVWS---AAGAGDVRSPCPALNTLANHNWLPHDGKNISKAALMSALTDGLNVGYDVALLLATGAFS------------- : 155
AaeAPO_Agr : AHPWK---PLRPGDIRGPCPGLNTLASHGYLPRNGVA-TPVQIINAVQEGLNFDNQAAVFATYAAHL------------- : 126
CfumagHTP_ : TLPYV---APGPTDSRAPCPALNALANHGYIPHDGRAISRETLQNAFLNHMGIANSVIELALTNAFV------------- :  77
MrotHTP1_M : AHPWK---APGPNDSRGPCPGLNTLANHGFLPRNGRNISVPMIVKAGFEGYNVQSDILILAGKIGML------------- :  65
HspHTP1_Hy : SSGWQ---APGPNDVRAPCPMLNTLANHGFLPHDGKGITVNKTIDALGSALNIDANLSTLLFGFAAT------------- :  87
MtherHTP_M : FDTWS---PPGPYDVRAPCPMLNTLANHGFLPHDGKDITREQTENALFEALHINKTLASFLFDFALT------------- :  82
                5           r pCP 6N 6anHg 6p  g
```

```
                      *       260         *       280         *       300         *       320
AglauHTP_A : ----------------MYHRVAPDAPFWTNFVAAPTIDLDMLAIHNLIEHDVSLTRLDSAL-------D--------DTA : 175
MuccirHTP_ : -----------------LQEANPHDSLFAQFKSLKTLDLDRLTVFGVLEHDVSLTRQDFAL-----GHQ--------DTS : 177
RdelHTP_Rh : -----------------YNEAVPDKHSLSQLGFTDKINLNQLSVYDVLEHDVSLTRHDTAL-----LPH--------NTT : 170
AbHTP1_Aga : -----------------KVVKKDY--------NKDTFNLDELDHHNGIEHDASLCREDSAL------CP--------DQS : 151
UmHTP3_Ust : -----------------VFSERAH-----------IFDLESIGIANIIEHDASLLRPDYHLADWHKDPK--------AMS : 271
GrosHTP2_G : -----------------LLGKLLDK----------TINLDDLNQHNKCEHDASLTRKDFYF-------G--------DNH : 118
PoxaHTP1_P : --------------HDETSHIGLRNPGQVNESGMPVLNLDQTGRPHAIEHDVSLSREDRAL-------G--------DCI : 124
GzHTP3_Gib : RSLLQKLWQTVTDPWSVLGKFGMRKPGQVDSEGHRVLNLDQLGLPNTVEHDISLTRRDHQQ-------G--------DNI : 151
PcarHTP4_P : -----------------LLGQGLG-----------PFELHDLARHNKIEHDASLFHPNIGP----REEY--------API : 109
SlutHTP4_S : ------------------FCGRAF-----------RLDLDALALHNKIEHDASLVHADALG-----QQR--------API : 112
SdicHTP1_S : -----------------LGHGG-------------LLTLADLDVHGAIEHDASLTRQDAAL-------G--------DNT : 127
SpispHTP_S : -----------------TGRETPS--------GK-VLDLDMLNEHNLIEHDASLTRSDSGV-------G--------DNS : 109
PparHTP1_P : -----------------------------------TIALSDLSQHNVWDHDASLVHTDAHF-------GH-------DPA : 139
PsojHTP2_P : -----------------------------------PINLDYLGIHNKIEHDASLAHTDAYY------VH--------DPM : 129
PfungHTP1_ : ------------------DLGTPE-----------TITLEELQKHNMIEHDASLSRLDNYL-------G--------DST : 191
AaeAPO_Agr : ----------VDGNLITDLLSIGRKTRLTGPDPPPPASVGGLNEHGTFEGDASMTRGDAFF-------G--------NNH : 181
CfumagHTP_ : --------------VCEYVTGSDC--------GDSLVNLTLLAEPHAFEHDHSFSRKDYKQ-------GVANSNDFIDNR : 128
MrotHTP1_M : -----------------TSREAD------------TISLEDLKLHGTIEHDASLSREDVAI-------G--------DNL : 101
HspHTP1_Hy : -----------------TNPQPNAT----------FFDLDHLSRHNILEHDASLSRQDSYF-------G--------PAD : 125
MtherHTP_M : -----------------TNPKNTS-----------TFSLNDLGNHNILEHDASLSRADAYF-------G--------NVL : 119
                                                   6         ehD S  r 1
```

```
                      *       340         *       360         *       380         *       400
AglauHTP_A : IPQANLVRRMHQWTL------QHDGQLDMQAEHDLRKIRWYESTLSNPKSHLGLLYQFSSSTECALLLDILGRD------ : 243
MuccirHTP_ : HVVPEYVDRMIKLAETKNQG-ELKGIFTRENENDARKLRWMESIRDNRHMHLNFFSQFASSTECSLLLDILGRD------ : 250
RdelHTP_Rh : YPAPELIERMIRLVELNNTSTENKEYFTFENEHDARKLRWLESTKHNPLFHYPLFLQIASSFECNLLLNTIGRH------ : 244
AbHTP1_Aga : KPHVEYITQLLAHATGKDK--DGNPLLTMKDLAKYSSKRRADARSYNKKFTLGFIHRMFGSSNSSTMLTIFGG------- : 222
UmHTP3_Ust : RPHQDMIDRWFPAPTKHG---KVKADLTDRDFAEAVCIRRSESRKHNRQYSSNVVQSLIGSGSCCLMLNVFGGKVLDLRE : 348
GrosHTP2_G : TVNPELVDLLLKQ--------NIDRKIKEEPLSKLHWIRLNNSKEVNPTLLYKFKQKFLSAGESSLLLNFIGAN------ : 184
PoxaHTP1_P : KADPDLVDKLIQYP-------GNKDSFTISDLGRFRKRRHTEQMARNSDLKFDSSKHKKACGEAALFQSIFGKG------ : 191
GzHTP3_Gib : TLQKDLVEDLLASS-------KDGKVITVDDLVEFRKKRIARQKEDNPGVQYGAFENDLACAEIALILNVIGTG------ : 218
PcarHTP4_P : HGEAQLLRAFLAMS-------ADGRVITPEDLARIRVQREAET-------PLDPLHAEIARGEMAIVFNMFNNPDPTLHE : 175
SlutHTP4_S : EVDPKLLKSFLSHA-------DPQRGMSLYDLAQVRISR--EAQLARP---LDLVHSQIGAAEAALCWLLLKQD------ : 174
SdicHTP1_S : KLDPTLLAAFQAIS-------SDGKYITKAELAAYRLARAADSKARNPAFRFGAREQVVAYAEAAVLFVAFED------- : 193
SpispHTP_S : AVNKTLVEQLLAFS-------KDGKTIDYDDMAKLRRLRYRQSQATNKNLIYGAWQYLLASGEAVLLIETFGGK------ : 176
PparHTP1_P : EVNITLANDLLNRG-------KASNKLGVNEVGNAYKDRRQAGKPYEPKYSLSDAQKTQSYSQGAGLLLVFGAK------ : 206
PsojHTP2_P : TANVTLAEEFFSRA-------GSDGLLTNSIVAKTRKDRGNTCNAQNPECSYGVQAQSLAFLEASVLLMALGSG------ : 196
PfungHTP1_ : KLDQTLFERLLNAS-------SDGITISIPDFVKYRRERQEESKTNNPSEVFGKKQLIAAAGEVVLVSTIFGSY------ : 258
AaeAPO_Agr : DFNETLFEQLVDYSNR-----FGGGKYNLTVAGELRFKRIQDSIATNPNFSFVDFRFFTAYGETTFPANLFVDG------ : 250
CfumagHTP_ : NFDAETFQTSLDVV-------AGKTHFDYADMNEIRLQRESLSNELDFPGWFTE-SKPIQNVESGFIFALVSDF------ : 194
MrotHTP1_M : HFNEAIFTTLANSN-------PGADVYNISSAAQVQHDRLADSLARNPNVTNTDLTATIRSSESAFFLTVMSAG------ : 168
HspHTP1_Hy : VFNEAVFNQTKSF--------WTGDIIDVQMAANARIVRLLTSNLTNPEYSLSDLGSAFSIGESAAYIGILGDK------ : 191
MtherHTP_M : QFNQTVFDETKTY--------WEGDTIDLRMAAKARLGRIKTSQATNPTYSMSELGDAFTYGESAAYVVVLGDK------ : 185
                                                   R
```

```
                           *        420         *        440         *        460         *        480
AglauHTP_A : ------------------------------GTMRADHIEALLLDERFP--DDWYPREKA--------------------- : 270
MuccirHTP_ : ------------------------------GTITLPHLKSFLLNESFP--EDWHPRETT--------------------- : 277
RdelHTP_Rh : ------------------------------GRLRVDHLESILLHERFP--NDWYPPTEP--------------------- : 271
AbHTP1_Aga : ---------------------------------RVKDLETVLLEERLP--ENWESRVRGKMG------------------ : 249
UmHTP3_Ust : MA---------------------GSVEHDL-PGVRTTRAGLEYGYERIPARTINAKKGSE--------------------- : 386
GrosHTP2_G : ----------------------TD------LEIEIEKLEVFLKHERFP--EGWRKPNKV--------------------- : 213
PoxaHTP1_P : ----------------------VF------YNLPTKYVKSMFQEERLPYEED----------------------------- : 215
GzHTP3_Gib : ------------------------------DRVECSYARAFLQQERLPIDEGWKKRS------------------------ : 245
PcarHTP4_P : AGVPLQPRRSIFRLVHTLLHRPDTGASRPLDGVPIDRLRYWFEHERIP--PGWEPYHE----------------------- : 231
SlutHTP4_S : ----------------------SG-------RIPSSTLLQWYGEERLP--DNWARPRRV---------------------- : 202
SdicHTP1_S : ----------------------DS------GAMRLDWLEMFFAQEKLPFELGWTLRH------------------------ : 222
SpispHTP_S : ----------------------EQ-------KIPIETARELLLHERFP--ENWTPKETP---------------------- : 204
PparHTP1_P : ----------------------NG------ESVSLDVARSFLVEERIP--DGWKPAATP---------------------- : 235
PsojHTP2_P : ------------------------------DSISVDHARSFIVDEKIP--ADYKAPKSS---------------------- : 223
PfungHTP1_ : ----------------------FS-------GLPVEAARTFFGSERFP--DGWTKPAFP---------------------- : 286
AaeAPO_Agr : ----------------------RRDD----GQLDMDAARSFFQFSRMP--DDFFRAPSPRSGTGVEVVIQAHPMQPGRNV : 302
CfumagHTP_ : ----------------------NLPDNDENPLVRIDWWKYWFTNESFPYHLGWHPPSPAREIEFVTSAS----------- : 241
MrotHTP1_M : ----------------------DPLR----GEAPKKFVNVFFREERMPIKEGWKRSTTP---------------------- : 201
HspHTP1_Hy : ----------------------KS------ATVPKSWVEYLFENERLPYELGFKRPNDP---------------------- : 222
MtherHTP_M : ----------------------ES------RTVKRSWVEWFFEHEQLPQHLGWKRPAAS---------------------- : 216
                                                   e  P
```

```
                       *       500         *       520         *       540         *       560
AglauHTP_A : ---------------------------YPAL--QALVRPLKCWYGIRKSAATLESLQALSNDTQQILV------------ : 309
MuccirHTP_ : ---------------------------FTLK--ELATKPLICWHGLRNSQVSLDLINELA-------------------- : 308
RdelHTP_Rh : ---------------------------TSLS--EILIDPLNCWKGLRSNVSFS--------------------------- : 295
AbHTP1_Aga : ---------------------------LTFAAFNLTVTPVELMISEKKYLKEKAAKEANKDD------------------ : 284
UmHTP3_Ust : ---------------------------CCPI--VSTTQKQRWQPATAKWYFGMTIFYALWTTFKIEMRARA--------- : 428
GrosHTP2_G : ---------------------------VGIW--PIISSLFSFLKRYDQLEKEQKK------------------------- : 239
PoxaHTP1_P : ------------------------------------SDIALCFANLI--------------------------------- : 226
GzHTP3_Gib : ---------------------------FGIV--SLITERDKVKKKLGMDFKS---------------------------- : 268
PcarHTP4_P : ---------------------------TTLL--DTVATSMRIRRAMKITREVASERRGRSH------------------- : 263
SlutHTP4_S : ---------------------------IGLL--DARSKAAEVTNIMSSLKKSTCRTTPSSSNFDVSVLGLRTILTCDS-- : 251
SdicHTP1_S : ---------------------------------ISLSRVLLVAGELRSSAFGAEVLAMIA-------------------- : 249
SpispHTP_S : ---------------------------TGRL--GLFANVAKLRFKMLTAPSHELEKQALRDEL----------------- : 238
PparHTP1_P : ---------------------------ITVA--DSRATSAKLREVAEAV------------------------------- : 255
PsojHTP2_P : ---------------------------VGVA--AVTARAALLKAQSLTWAM----------------------------- : 245
PfungHTP1_ : ---------------------------VFAA--ELIPQITRVAWQCS--------------------------------- : 304
AaeAPO_Agr : GKINSYTVDPTSSDFSTPCLMYEKFVNITVK--SLYPNPTVQLRKALNTNLDFFFQGVAAGCTQVFPYGRD--------- : 371
CfumagHTP_ : ---------------------------SAVLAASVTSTPSSLPSGAIGPGAEAVPLSFASTMTPFLLATNAPYYAQDPTL : 294
MrotHTP1_M : ---------------------------ITI---PLLGPIIERITELSDWKPTGDNCGAIVLSPEL--------------- : 236
HspHTP1_Hy : ---------------------------FTTDDLGDLSTQIINAQHFPQSPGKVEKRGDTRCPYGYH-------------- : 261
MtherHTP_M : ---------------------------FEEE--DLNSSMEEIEKYTKELEGSNSTSGSQKHRRRLPRRRAHFGF------ : 261
```

```
AglauHTP_A : ---- :   -
MuccirHTP_ : ---- :   -
RdelHTP_Rh : ---- :   -
AbHTP1_Aga : ---- :   -
UmHTP3_Ust : ---- :   -
GrosHTP2_G : ---- :   -
PoxaHTP1_P : ---- :   -
GzHTP3_Gib : ---- :   -
PcarHTP4_P : ---- :   -
SlutHTP4_S : ---- :   -
SdicHTP1_S : ---- :   -
SpispHTP_S : ---- :   -
PparHTP1_P : ---- :   -
PsojHTP2_P : ---- :   -
PfungHTP1_ : ---- :   -
AaeAPO_Agr : ---- :   -
CfumagHTP_ : GPND : 298
MrotHTP1_M : ---- :   -
HspHTP1_Hy : ---- :   -
MtherHTP_M : ---- :   -
```
